# Mesoporous WO_3_ Nanofibers With Crystalline Framework for High-Performance Acetone Sensing

**DOI:** 10.3389/fchem.2019.00266

**Published:** 2019-04-18

**Authors:** Haiyun Xu, Jie Gao, Minhan Li, Yuye Zhao, Ming Zhang, Tao Zhao, Lianjun Wang, Wan Jiang, Guanjia Zhu, Xiaoyong Qian, Yuchi Fan, Jianping Yang, Wei Luo

**Affiliations:** ^1^State Key Laboratory for Modification of Chemical Fibers and Polymer Materials, College of Materials Science and Engineering, Donghua University, Shanghai, China; ^2^Materials Genome Institute, Shanghai University, Shanghai, China; ^3^Institute of Functional Materials, Donghua University, Shanghai, China; ^4^School of Materials Science and Engineering, Jingdezhen Ceramic Institute, Jingdezhen, China

**Keywords:** mesoporous materials, WO_3_, nanofibers, electrospun, acetone, sensor

## Abstract

Semiconducting metal oxides with abundant active sites are regarded as promising candidates for environmental monitoring and breath analysis because of their excellent gas sensing performance and stability. Herein, mesoporous WO_3_ nanofibers with a crystalline framework and uniform pore size is successfully synthesized in an aqueous phase using an electrospinning method, with ammonium metatungstate as the tungsten sources, and SiO_2_ nanoparticles and polyvinylpyrrolidone as the sacrificial templates. The obtained mesoporous WO_3_ nanofibers exhibit a controllable pore size of 26.3–42.2 nm, specific surface area of 24.1–34.4 m^2^g^−1^, and a pore volume of 0.15–0.24 cm^3^g^−1^. This unique hierarchical structure, with uniform mesopores and interconnected channels, could facilitate the diffusion and transportation of gas molecules in the framework. Gas sensors, based on mesoporous WO_3_ nanofibers, exhibit an excellent performance in acetone sensing with a low limit of detection (<1 ppm), short response-recovery time (24 s/27 s), a linear relationship in a broad range, and good selectivity.

## Introduction

Over the past few decades, the precise monitoring of toxic polluting gases has attracted great attention in modern society in environmental protection, industrial production, health care, and so forth (Kawano et al., 2007; Wang et al., 2007; Salehi et al., 2014). Acetone is a common type of reagent which is frequently used in manufacturing industries and laboratories (Zhang et al., 2017). As a highly toxic gas, acetone is harmful to human health, and long-term exposure to acetone may cause irritation and damage to the eyes, nose, and central nervous system (Jia et al., 2014). In addition, acetone concentrations in respiration can be characterized as a biomarker in the rapid diagnosis of diabetes, because acetone concentrations exhaled from diabetes patients (1.8 ppm) are much higher than in a healthy individual (0.3–0.9 ppm) (Singkammo et al., 2015; Zhou et al., 2018). Therefore, it is of great interest and importance to develop acetone sensors with a low detection limit, fast response recovery dynamics, high sensitivity and selectivity. Among a variety of sensing-active materials used for the detection of acetone, metal oxide semiconductors (WO_3_, TiO_2_, SnO_2_, ZnO, Fe_2_O_3_, etc.) have been considered as appealing candidates due to their remarkable sensitivity, low cost, and excellent stability (Wang et al., 2010; Luo et al., 2013; Kim and Lee, 2014; Zhu et al., 2017a; Zhou et al., 2018). The sensing mechanism of semiconducting metal oxides (SMOs)-based sensors is widely accepted to be the change in conductivity when exposed in specific gas. The adsorbed gaseous analytes may cause an increase or decrease in the resistance of semiconducting metal oxides (Barsan and Weimar, 2001; Yamazoe et al., 2003; Qiu et al., 2018; Zhang Q. et al., 2018). Given that the gas-sensing process involves the adsorption–desorption and catalytic reactions on the surface of the metal oxides, a rational design and controllable synthesis of nanomaterials with high surface areas, abundant active sites, tailor-designed nanostructures and outstanding catalytic performance, are considered as promising approaches to enhance the sensing performance of the semiconducting metal oxides.

Mesoporous semiconducting metal oxides (MSMOs), as an important category of nanostructured materials, have drawn much attention because of their high surface area, uniform pore size, highly crystalline framework, numerous active sites, interconnected pore structure and large pore size. The high surface area and abundant active sites greatly facilitates the interaction between metal oxide frameworks and gaseous molecules, as well as surface catalytic reactions. In addition, the large porosity and well-connected mesostructure favors rapid and effective diffusion of gas molecules (Li et al., 2014; Luo et al., 2016b; Ma et al., 2018). Therefore, MSMOs are regarded as promising candidates for gas sensing. To date, various MSMOs have been synthesized through different approaches, such as sol–gel processes, spray pyrolysis, chemical vapor deposition and precipitation reactions (Du et al., 2011; Luo et al., 2016a; Zhao et al., 2016; Channei et al., 2018; Jha G. et al., 2018; Zhang Y. et al., 2018). However, these methods usually give rise to an uncontrolled morphology and low porosity, which is not favorable for sensing performance. Additionally, a lot of work has been focused on the design and construction of various nanostructures of SMOs to improve the their sensing performance, including zero-dimensional nanoparticles (NPs) (Yang Z. et al., 2018; Zhang H. et al., 2018; Zhao et al., 2019), one-dimensional nanofibers (NFs) (Saha and De, 2013; Kim et al., 2016a; Ren et al., 2016; Nada et al., 2017; He et al., 2018; Jeong et al., 2018) and nanowires (NWs) (Wang et al., 2004; Rakhi et al., 2012; Dam and Lee, 2013; Chen et al., 2015; Li X. et al., 2017), two-dimensional nanosheets (Wang et al., 2016, 2017; Li F. et al., 2017; Kaneti et al., 2018) and membranes (Dasog et al., 2012; Barr et al., 2017; Jha G. et al., 2018; Wang W. Q. et al., 2018). Nanofibers have drawn particular attention due to their exceptionally high surface area-to-volume ratio, high porosity, superior surface permeability and accessibility, making them an attractive candidate for gas sensing (Guo et al., 2014; Jha R. K. et al., 2018; Yan et al., 2018). Electrospinning technology has been demonstrated as an effective approach to prepare micro-sized and nano-sized fibers. The structure, morphology and dispersion of functional components of the fibers synthesized by electrospinning can be well-tailored through well-controlled conditions and compositions (Wang and Hashimoto, 2018; Yoon et al., 2018; Zhang D. et al., 2018). Kim et al. fabricated semiconducting metal oxide nanofibers through a protein nanocage templating route, to detect trace amounts of target biomarkers in exhaled breath (Kim et al., 2016a). Nevertheless, up to now, it remains a great challenge to construct mesoporous structures in nanofibers with controllable pore size and morphologies, which is highly desirable for improved gas sensing performance.

Among various SMOs, tungsten oxide (WO_3_), an *n*-type semiconductor with a band-gap of 2.5 eV, is a promising sensing material for the detection of gas due to its variable oxidation states and suitable band structure (Wang C. Y. et al., 2018). Herein, we adopt a facile approach based on electrospinning to synthesize mesoporous WO_3_ nanofibers (NFs) with uniform and controllable pore sizes using SiO_2_ nanoparticles and polyvinylpyrrolidone (PVP) as sacrificial templates, ammonium paratungstate as a tungsten precursor and water as a solvent. Due to the support of the rigid PVP species during electrospinning process, the interconnected porous structure and unique fiber-like morphology can be well-maintained after calcination in nitrogen and air, followed by treatment with hydrofluoric acid selectively etched silica particles, creating uniform mesopores in the nanofibers. The obtained mesoporous WO_3_ NFs have crystalline frameworks, large uniform pore sizes of 26.3 nm and 42.2 nm, and their specific surface area and pore volume can be as high as 34.4 m^2^/g and 24.1 cm^3^/g, respectively. Moreover, the mesoporous WO_3_ NFs based sensors exhibit superior gas sensing performance with a fast response (24 s) and recovery (27 s), high sensitivity of 23 (*R*_a_/*R*_g_) at 50 ppm when operating at 300°C, an ultralow limit of detection of 1 ppm, and good selectivity, which contributes to their good merits of suitable pore size, high surface area and abundant active sites located on the surface, and continuous and crystalline framework with open pore channels. Such an excellent sensing performance opens up the possibility for the mesoporous WO_3_ NFs based sensor to be used in many fields such as environmental monitoring and in the rapid diagnosis of disease.

## Experimental Section

### Chemicals and Materials

Tetraethyl orthosilicate (TEOS), ethanol, NH_4_OH solution and hydrofluoric acid of AR grade were purchased from Sino-Pharm Chemical Reagent Co. Ltd. Polyvinylpyrrolidone (PVP, M_w_ ≈ 40,000 g/mol) was purchased from Aldrich. Ammonium metatungstate hydrate [(NH_4_)_6_H_2_W_12_O_40_·xH_2_O, 99.5% metals basis] was purchased from Aladdin.

### Preparation of SiO_2_ NPs

The SiO_2_ NPs with different diameters was prepared using a base catalyzed sol-gel method previously reported (Dasog et al., 2012). Typically, TEOS (10 mL) was dissolved in a solvent mixture of ethanol (10 mL), deionized water (20 mL) and NH_4_OH solution (42 %, 5 mL). The obtained solution was further stirred for 1 or 2 h to yield different size SiO_2_ NPs. The white precipitate was collected by vacuum filtration and washed with deionized water four times. The solid was transferred to an oven and was kept there for 24 h at 100°C to remove any residual water and ethanol.

### Synthesis of Mesoporous WO_3_ NFs

Typically, mesoporous WO_3_ NFs were synthesized through an electrospinning method as follows: (NH_4_)_6_H_2_W_12_O_40_·xH_2_O (2.7 g) and PVP (3.0 g) was dissolved in deionized water (4 mL). This was followed by the addition of SiO_2_ NPs (0.035 g) with a diameter of 25 nm. Then, the solution was stirred for 20 h for further electrospinning. The as-prepared gel was loaded into a plastic syringe and connected to a high voltage power supply for electrospinning. Twenty kilovolts high voltage was applied between the spinneret and the collector in a gap of 15 cm. In this way, hybrid precursor nanofibrous membranes were obtained. Then, the as-made products were heated with a ramp of 1°C min^−1^ to 350°C for 3 h in nitrogen, resulting in the carbon-supported amorphous tungsten oxide powders. The carbon species was removed and crystallization of the amorphous tungsten oxide frameworks was carried by subsequent heat treatment with a ramp of 1°C min^−1^ to 500°C in air for another 1 h. SiO_2_ NPs was completely removed from the composites by treatment with a HF solution (5 wt% aqueous solution, 8 mL) for 2 h and subsequently washed with deionized water and ethanol. Finally, the obtained sample was denoted as mesoporous WO_3_-25 NFs. Through the same approach, silica NPs with larger diameters (~40 nm) were also used as a hard template to synthesize mesoporous WO_3_ NFs, and the obtained samples were denoted as mesoporous WO_3_-40 NFs. Control experiments, without addition of SiO_2_ NPs, were performed according to the same method and procedure, and the obtained sample was denoted as a non-mesoporous WO_3_ NFs.

### Measurement and Characterization

Field-emission scanning electron microscopy (FE-SEM) was operated on a Hitachi S4800 (Japan) field-emission scanning electron microscope. Transmission electron microscopy (TEM) was conducted on a JEM-2100 F at an accelerating voltage of 200 kV. Wide-angle X-ray diffraction (XRD) patterns were collected on a Rigaku D/Max-2550 PC diffractometer (Tokyo, Japan) in the 2θ range of 10–90°. Nitrogen sorption isotherms were measured at 77 K with a Micromeritics Tristar 3020 analyzer (USA). Before measurements, the samples were degassed under vacuum at 180°C for at least 6 h. The Brunauer-Emmett-Teller (BET) method was utilized to calculate the specific surface areas using the adsorption data at *P*/*P*_0_ = 0.02–0.20. The pore size distribution (PSD) was calculated from the adsorption branch using the Barrett-Joyne-Halenda (BJH) model. The total pore volume (V_total_) was estimated from the adsorbed amount at *P*/*P*_0_ = 0.995. The XPS were collected on an RBD 147 upgraded PHI 5000C ESCA system with a dual X-ray source. The Mg Kα (1253.6 eV) anode and a hemispherical energy analyzer were used in the measurements. All of the binding energies were referenced to the C 1s peak at 284.8 eV of the surface adventitious carbon.

### Gas Sensing Performance

**Figure 1:**
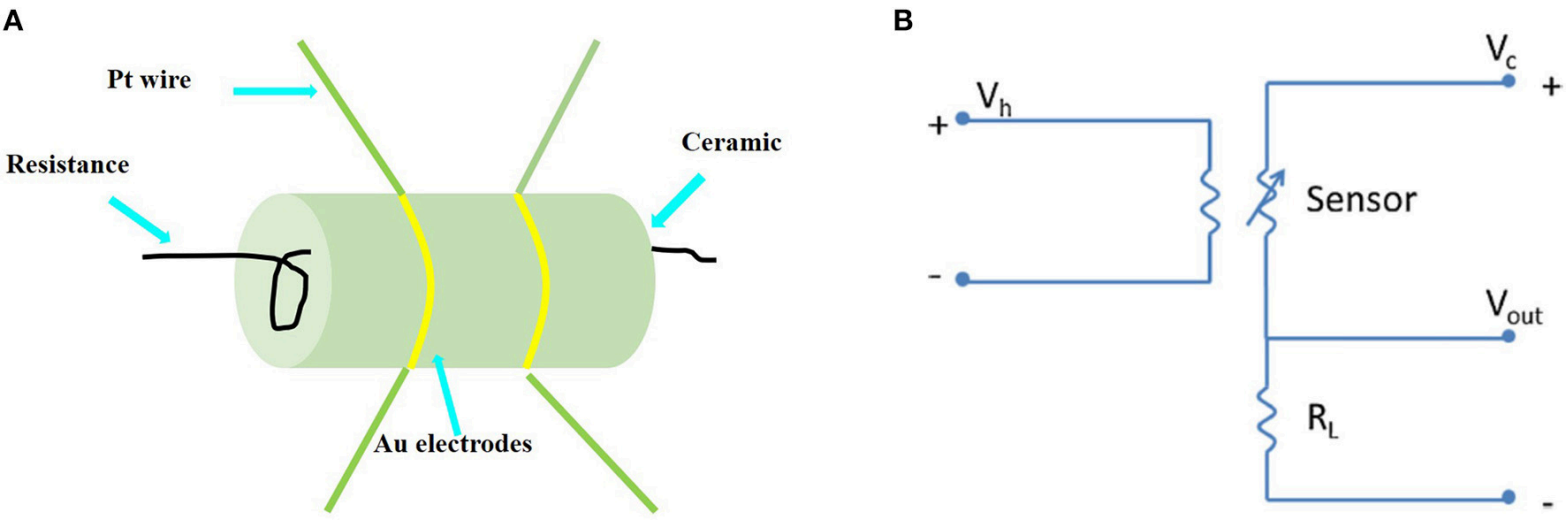
**(A)** Sketch of the structure of the side-heated mesoporous WO_3_ NFs based gas sensor. **(B)** Electric circuit of gas sensing measurements.

Side-heated gas sensors were prepared using a similar method reported previously (Zhang et al., 2009). The mesoporous (or non-mesoporous) WO_3_ NFs was mixed with deionized (DI) water and ground in an agate mortar to form a paste. The mass ratio of mesoporous (or non-mesoporous) WO_3_ NFs to DI water in the paste is 7:3. The paste was coated on an alumina ceramic tube printed with a pair of Au electrodes, and the thickness of the paste membrane was about 300 μm. Subsequently, the coated alumina ceramic tube was dried at room temperature for 24 h and then annealed at 300°C for 2 h with a ramping rate of 5°C/min in air. Then, a Ni–Cr alloy wire was inserted into the tube as a heater, and the working temperature could be adjusted by changing the heating voltage. Furthermore, the obtained sensor was kept at the optimal working temperature for a week before measurement to further improve the long-term stability. The assembled sensing device is depicted in Figure 1A. The stationary state gas distribution method was applied for testing the gas response. In the electric circuit for measuring the gas response (Figure 1B), a load resistor (*R*_L_) was connected in series with a gas sensor. The circuit voltage (*V*_C_) was set at 5 V and the output voltage (*V*_OUT_) was the terminal voltage of the load resistor. Test gases (such as acetone, methanol, ethanol, toluene, formaldehyde) were injected into a test chamber and diluted with air. The gas response of the sensor is defined as *S* = *R*_a_/*R*_g_ (for reducing gases) or *S* = *R*_g_ /*R*_a_ (for oxidizing gases), where *R*_a_ is the sensor resistance in air and *R*_g_ is that in the gas tested. The response time is defined as the time taken for the variation in conductance to reach 90% of the equilibrium value after a test gas was injected, and the recovery time is the time taken for the sensor to return to 10% above the original conductance in air after releasing the test gas, respectively.

## Results and Discussion

**Scheme 1:**
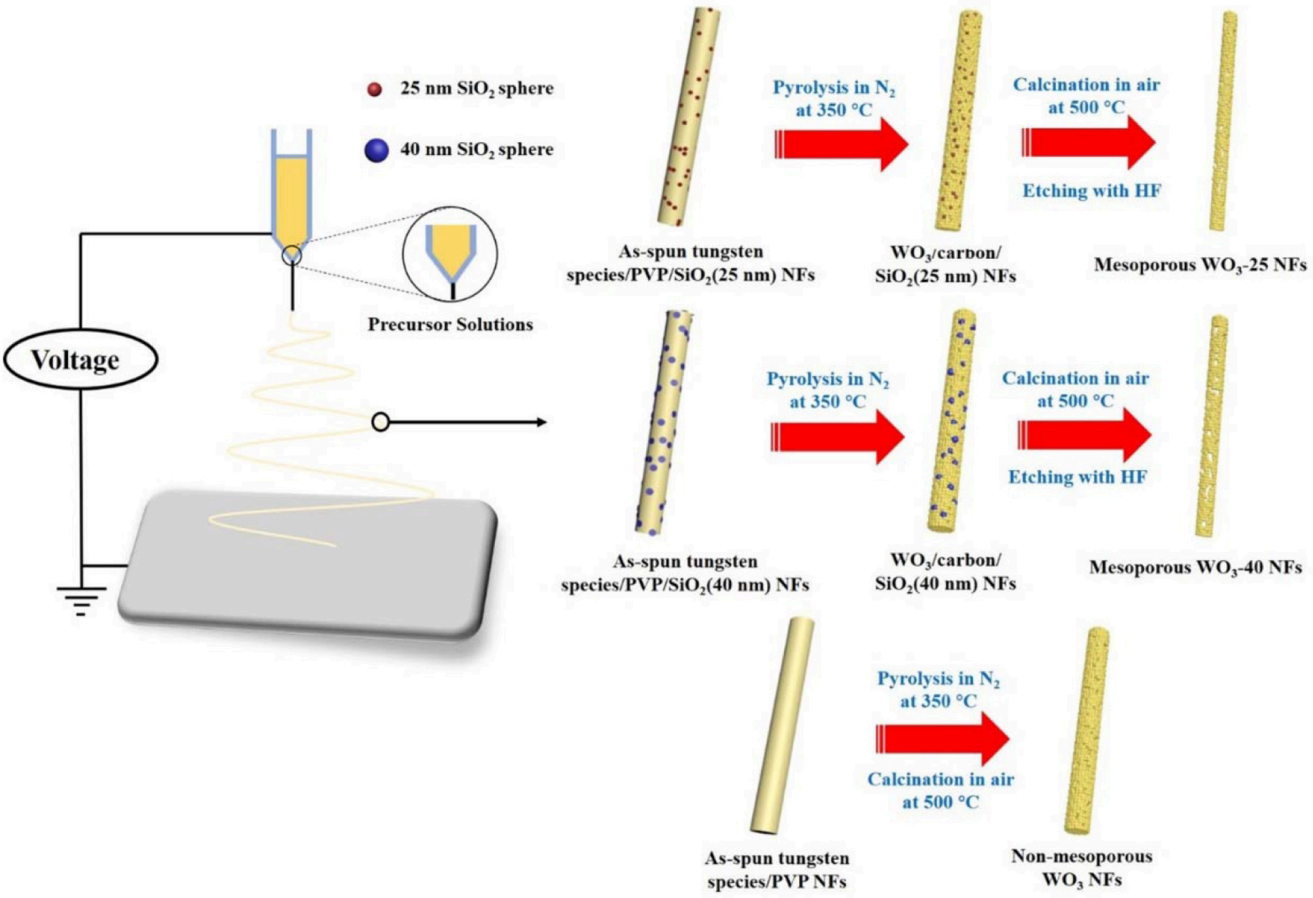
The fabrication process of the mesoporous WO_3_ NFs by electrospinning approach.

The diameters of the as-synthesized SiO_2_ NPs are estimated to be 25 and 40 nm from the TEM images (Figures S1A,B). An electrospinning technique was employed to construct mesoporous WO_3_ NFs from precursor solutions containing (NH_4_)_6_H_2_W_12_O_40_·*x*H_2_O, PVP, SiO_2_ NPs, and deionized water, as shown in Scheme 1. The as-spun tungsten species/PVP/SiO_2_ NFs was subjected to calcination at 350°C in nitrogen, giving rise to WO_3_/carbon/SiO_2_ NFs owing to the partial carbonization of the PVP species. The carbonized PVP can provide carbon support inside the frameworks, which can prevent the collapse of the fiber morphology during the crystallization process of WO_3_. Finally, after further pyrolysis at 500°C in air, and selective etching with hydrofluoric acid to remove the supporting carbon species and SiO_2_ sacrificing templates, mesoporous WO_3_ NFs is formed as a result. By using SiO_2_ NPs with different diameters, two WO_3_ NFs can be obtained and denoted as mesoporous WO_3_-*x* NFs, wherein *x* represents the particle size of SiO_2_ NPs. For comparison, non-mesoporous WO_3_ NFs was also synthesized via the same approach but without SiO_2_ NPs loading.

**Figure 2:**
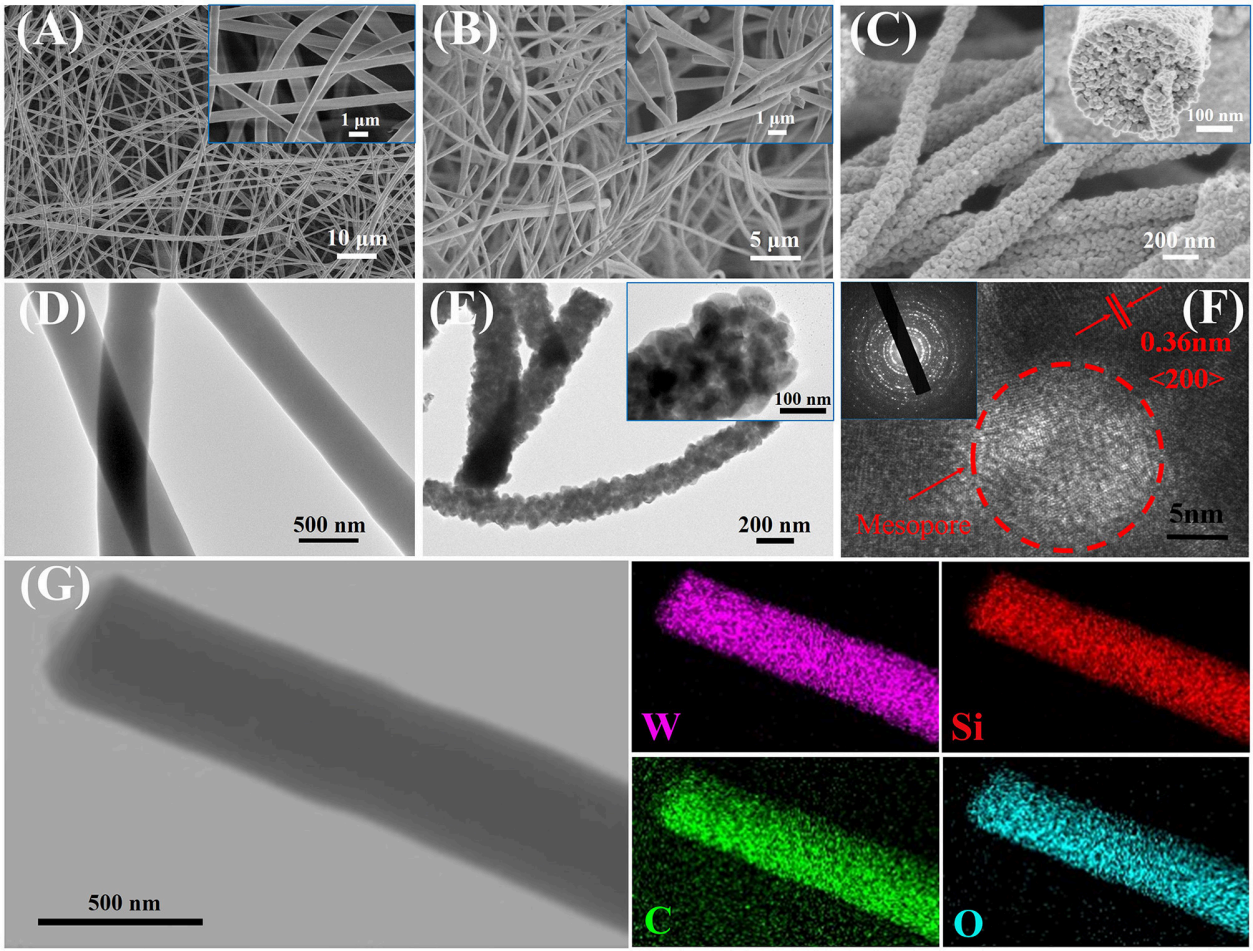
FESEM images of **(A)** as-spun tungsten species/PVP/SiO_2_ (25 nm) NFs, **(B)** WO_3_/carbon/SiO_2_ (25 nm) NFs, **(C)** mesoporous WO_3_-25 NFs. TEM images of **(D)** WO_3_/carbon/SiO_2_ (25 nm) NFs, **(E)** mesoporous WO_3_-25 NFs. HRTEM image of **(E)** mesoporous WO_3_-25 NFs. **(F)** Scanning TEM image and the corresponding EDS mapping images **(G)** of elemental W, Si, C, and O for WO_3_/carbon/SiO_2_ (25 nm) NFs.

Field-emission scanning electron microscopy (FE-SEM) observation reveals that all of the as-spun composites have a uniform fiber-like morphology with a dimeter of about 450–500 nm (Figure 2A; Figures S2A, S3A) and smooth surfaces (insets in Figure 2A; Figures S2A, S3A). After pyrolysis in N_2_ at 350°C, black WO_3_/carbon /SiO_2_ (25 nm), WO_3_/carbon/SiO_2_ (40 nm), and WO_3_/carbon NFs can be obtained, owing to the partially carbonization of the PVP species. Compared to the as-spun NFs, the composites retained their uniform fiber morphology, and much rougher surfaces are clearly visible in the surface (Figure 2B; Figures S2B, S3B). After complete removal of carbon and silica species by further calcination in air at 500°C and etching with hydrofluoric acid, all the WO_3_ NFs also retained the fiber morphology, indicating a good thermal stability, and their diameters were decreased to 250–300 nm due to the framework shrinkage at high temperature (Figure 2C; Figures S2C, S3C). It is worth noting that the WO_3_ NFs consisted of large numbers of nanograins, and boundaries can be clearly observed. An FESEM image taken along the cross-section of the mesoporous WO_3_-25 NFs clearly indicates the existence of mesopores around 25–30 nm (inset in Figure 2C), which is in good agreement with the diameter of SiO_2_ nanoparticles. Due to a larger diameter of the sacrificial SiO_2_ template, uniform mesopores of about 40–50 nm can be clearly visible in the SEM image of mesoporous WO_3_-40 NFs (Figure S2C). And no obvious mesopores were observed on non-mesoporous WO_3_ NFs (Figure S3C), attributing to the absence of the sacrificial SiO_2_ template.

Transmission electron microscopy (TEM) observations further confirmed that the NFs obtained after pyrolysis at 350°C are fibrous in shape with a diameter of 400–450 nm (Figure 2D; Figures S2D, S3D). The scanning TEM (STEM) image and energy dispersive X-ray elemental mapping recorded on WO_3_/carbon/SiO_2_ (25 nm) NFs clearly reveals that the W, Si, C, and O elements are homogeneously distributed in entire NFs (Figure 2G). It suggests that the carbonized PVP molecules can provide rigid support inside the frameworks, forming a “reinforced-concrete” framework structure with WO_3_ species. which can prevent the collapse of the NFs. TEM images of mesoporous WO_3_-25 and mesoporous WO_3_-40 NFs clearly indicate that the fiber-like morphology are well-retained, and the material consists of interconnected WO_3_ nanoparticles, as well as the existence of numerous mesopores (Figure 2E; Figure S2E), which can offer abundant active sites to interact with guest molecules, greatly facilitating the diffusion of gas molecules. Additional EDS analyses of the mesoporous WO_3_-25 NFs and WO_3_-40 NFs were carried out as depicted in Figures S4A,B, respectively. The absence of peaks from Si in the EDS spectrum indicates the complete removal of SiO_2_ by HF etching. Similarly, no obvious mesopores, but only WO_3_ nanoparticles, were shown in the TEM image of non-mesoporous WO_3_ NFs (Figure S3E). High resolution TEM (HR-TEM) images of mesoporous and non-mesoporous WO_3_ NFs clearly show the lattice fringes of tungsten oxide with a *d*-spacing of 0.36 nm (Figure 2F; Figures S2F, S3F), corresponding to the (200) planes, suggesting that the framework consists of well-crystallized and interconnected WO_3_ nanoparticles. Selected-area electron diffraction (SAED), recorded on different WO_3_ NFs showed well-resolved diffraction rings corresponding with the (200), (112), and (022) crystal planes of monoclinic phase WO_3_ (insets in Figure 2F, Figures S2F, S3F), further confirming the crystalline feature of the scaffold.

**Figure 3:**
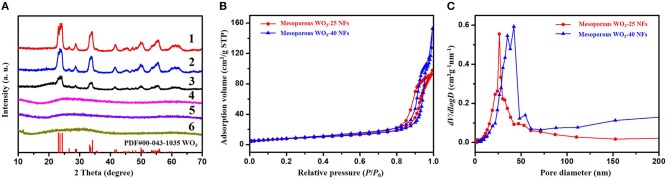
**(A)** XRD patterns of (1) mesoporous WO_3_-25 NFs, (2) mesoporous WO_3_-40 NFs, (3) non-mesoporous WO_3_ NFs, (4) WO_3_/carbon/SiO_2_ (25 nm) NFs, (5) WO_3_/carbon/SiO_2_ (40 nm) NFs, (6) WO_3_/carbon NFs. **(B)** Nitrogen-sorption isotherms and **(C)** pore size distribution curves of mesoporous WO_3_-25 NFs and mesoporous WO_3_-40 NFs.

Wide-angle X-ray diffraction (WA-XRD) patterns indicate that the WO_3_/carbon/SiO_2_ (25 nm), WO_3_/carbon/SiO_2_ (40 nm), and WO_3_/carbon NFs exbibits the amorphous feature contributing to the poor crystallization (Figure 3A). After crystallization at 500°C, and removal of the silica nanoparticles by selective etching with hydrofluoric acid, mesoporous WO_3_-25, mesoporous WO_3_-40 and non-mesoporous WO_3_ NFs shows well-resolved diffraction peaks in the range of 10–70°C, matching well with the crystalline monoclinic phase of WO_3_ with lattice parameters of a = 0.7297, b = 0.7539, c = 0.7688 nm, and β = 90.91 (JCPDS No. 43–1,035). No diffraction peaks from other crystalline impurities are observed in the XRD patterns, indicating pure crystalline phase, which agreeing well with the HR-TEM results. The broadening of the diffraction peaks can be attributed to the small particle size of WO_3_ nanocrystals (Li et al., 2014).

Nitrogen adsorption-desorption isotherms of the obtained mesoporous WO_3_-25 NFs exhibit type-IV curves with H1 hysteresis loop (Figure 3B). The steep increase in the adsorption band at *P*/*P*_0_ = 0.75–0.96 indicates mesopores with a large and uniform pore size. The pore diameter is about 26.3 nm as indicated in the pore size distributions derived from the adsorption branch of the isotherms by using Barrett-Joyner-Halenda (BJH) model (Figure 3C). The specific surface area and total pore volume of mesoporous WO_3_-25 NFs are calculated to be as high as 34.4 m^2^g^−1^ and 0.15 cm^3^g^−1^, respectively. Similarly, mesoporous WO_3_-40 NFs also display type-IV curves with sharp capillary condensation steps in the relative pressure range of 0.78–0.97 (Figure 3B). The pore size distribution profile reveals a mesopore size of 42.2 nm (Figure 3C), indicating that the hard template silica nanoparticles with larger diameters result in larger mesopores. The surface area and total pore volume of mesoporous WO_3_-40 NFs are approximately 24.1 m^2^g^−1^ and 0.24 cm^3^g^−1^, respectively. Such a porous structure provides an amplified target-receptor interface and is beneficial for the diffusion and adsorption of large guest molecules, making it an ideal candidate for many applications involving host-guest interactions, such as catalysis and gas sensing. In addition, nitrogen adsorption–desorption isotherms of non-mesoporous WO_3_-NFs show type-IV curves with H3 hysteresis loop (Figure S5A). The surface area and total pore volume of non-mesoporous WO_3_NFs is 19.5 m^2^g^−1^ and 0.17 cm^3^g^−1^, respectively. The average pore size calculated from the adsorption branch using the BJH method is about 5.3 nm (Figure S5B), much smaller than that of the etched samples (26.3 nm for mesoporous WO_3_-25 NFs and 42.2 nm for mesoporous WO_3_-40 NFs), which can be attributed to the stacked pores of small WO_3_ grains.

X-ray photoelectron spectroscopy (XPS) is further used to investigate the surface composition and elemental states of the NFs before and after etching. For the WO_3_/SiO_2_ (25 nm) composite NFs (denoted as WO_3_-25/SiO_2_ NFs) before etching, four peaks corresponding to W 4f, Si 2p, W 4d and O 1s are shown in the survey spectrum (Figure S6A). However, after etching, the Si 2p peak at 103.3 eV disappears, indicating the complete removal of SiO_2_ species by HF (Xu et al., 2015; Shi et al., 2016). The high-resolution XPS W 4d spectrum (Figure S6B) of the samples after etching show similar spectra and can be fitted with two peaks centering at 35.8 and 37.9 eV, which can be assigned to W^6+^ 4f_7/2_ and W^6+^ 4f_5/2_, respectively (Wang et al., 2012). These results indicate that completely oxidized W is not changed during the etching process in mesoporous WO_3_-25 NFs, mesoporous WO_3_-40 NFs and non-mesoporous WO_3_ NFs samples. The state of O 1s indicates two types of oxygen in the surface (Figure S6C), lattice oxygen (O^2−^) and adsorbed oxygen (O^−^ and O^2−^). Usually, the adsorbed oxygen was more active to react with reducing gases compared with lattice oxygen, changing the concentration of main carriers (Zhu et al., 2017a).

**Figure 4:**
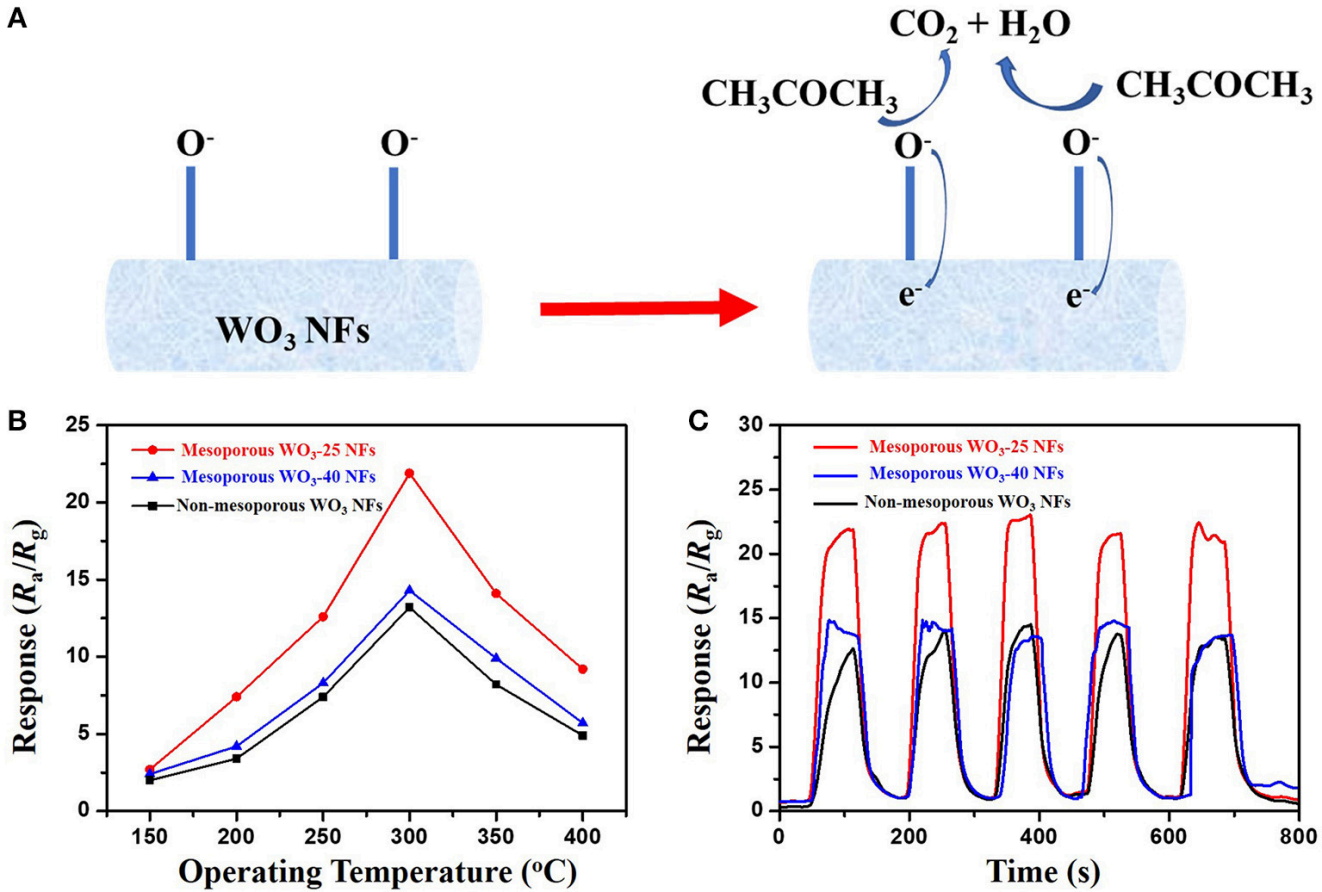
**(A)** sensing mechanism of WO_3_ NFs toward acetone. **(B)** responses of the mesoporous and non-mesoporous WO_3_ NFs sensor to 50 ppm acetone at different operating temperatures (150–400°C). **(C)** repeating response and recovery curve of the mesoporous and non-mesoporous WO_3_ NFs to 50 ppm acetone.

Inspired by the unique structure of obtained mesoporous WO_3_ fibers with ultra-large and controllable pore size, we tested the performance of mesoporous WO_3_-25, mesoporous WO_3_-40 and non-mesoporous WO_3_ NFs as sensing materials for the detection of acetone to investigate their potential application in the detection of acetone leakage and diagnosis for diabetes. The schematic diagram of sensing mechanism is shown in Figure 4A. In the gas sensing test, probe gases such as acetone were injected into a test chamber and mixed with air. The gas response of the sensor in this study is defined as *S* = *R*_a_/*R*_g_, where *R*_a_ and *R*_g_ is the resistance of the sensor in air and test gas, respectively. The response time is defined as the time required from *R*_a_ to *R*_a_-90% × (*R*_a_-*R*_g_) after a test gas was injected, and the recovery time is defined as the time required from *R*_g_ to *R*_g_ + 90% × (*R*_a_-*R*_g_) in air after releasing the test gas, respectively. Since the sensing performances of semiconductors for a specific gas are usually dependent on the working temperature, parallel tests of mesoporous WO_3_-25, mesoporous WO_3_-40 and non-mesoporous WO_3_ NFs based sensors were carried out toward a 50 ppm acetone gas in a range of 150–400°C to optimize the working temperature region (Figure 4B). It can be seen that the response of all sensors increased continuously until reaching a maximum value at 300°C and then decreased upon increase of the operating temperature. Such response behavior is due to the balance of the competition between the increase of the surface reaction rates and the decrease in the number of active sites for the adsorption of acetone at high temperatures. As a result, 300°C was adopted as the optimum working temperature for subsequent acetone detections.

**Figure 5:**
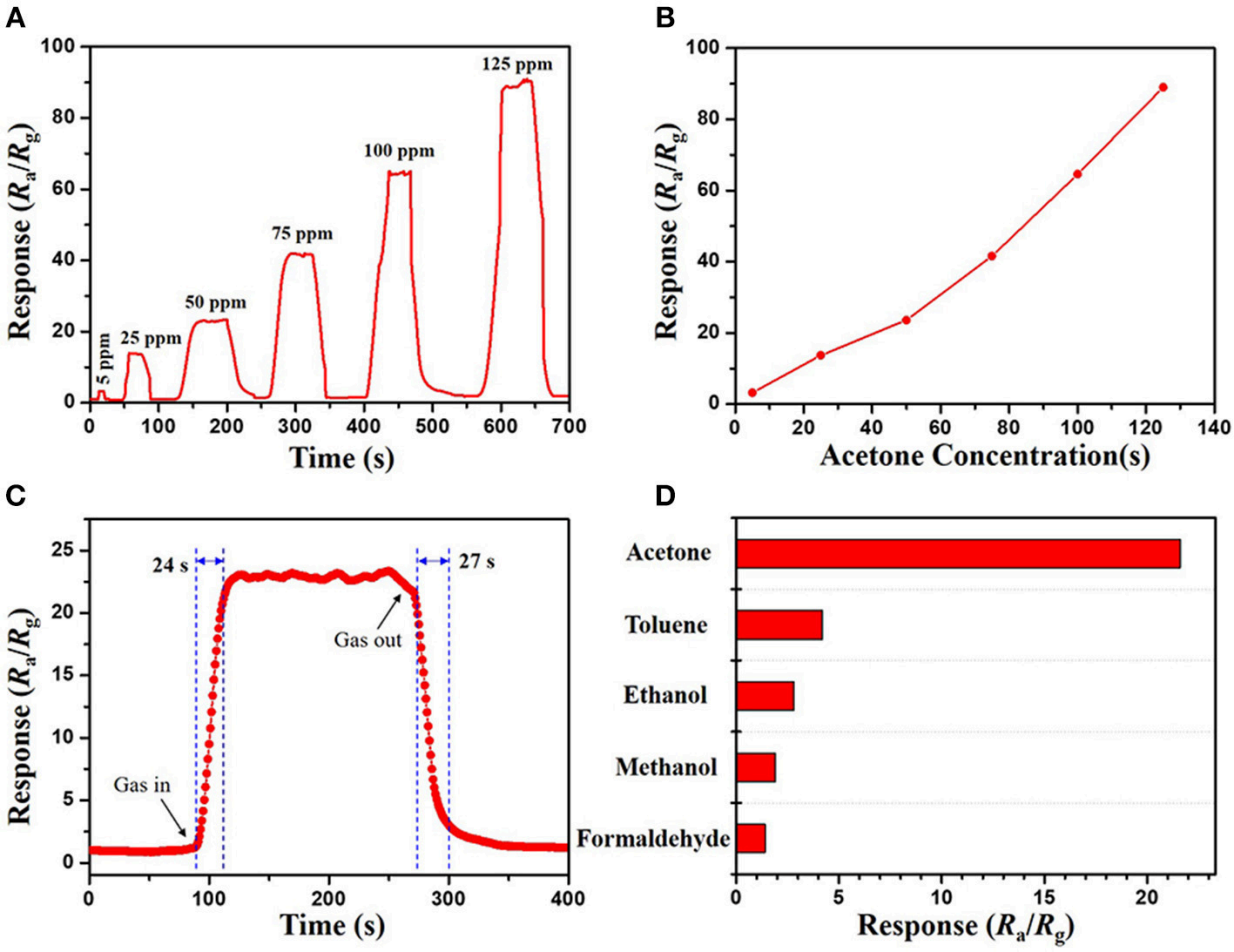
**(A)** response–recovery curve and **(B)** relationships between acetone concentration and response of the mesoporous WO_3_-25 NFs sensor to acetone vapors of different concentrations. **(C)** dynamic response–recovery curve of mesoporous WO_3_-25 NFs sensor to 50 ppm acetone. **(D)** responses of the mesoporous WO_3_-25 NFs sensor to various gases at 50 ppm.

In contrast, it was found that both of the mesoporous sensing materials, mesoporous WO_3_-25 and WO_3_-40 NFs, have shown higher responses to acetone than the non-mesoporous WO_3_ NFs. Such phenomenon may contribute to the uniform mesopores and interconnected transportation channels of mesoporous WO_3_ NFs resulting from the sacrificial templates including silica nanoparticles and PVP species, which provides a huge interface for the creation of active sites for acetone gas interaction, and greatly facilitates the diffusion of gas molecules in the framework. In addition, the response value increases dramatically from 13.7 for mesoporous WO_3_-40 NFs to 22.1 for mesoporous WO_3_-25 NFs at the same temperature, indicating that the sensitivity of the materials is closely related to its specific surface area. Mesoporous WO_3_-25 NFs with a higher surface area (34.4 m^2^g^−1^) could provide more active surface sites for numerous surface reactions between guest molecules and adsorbed oxygen species on the solid-gas interface. Therefore, the response value of mesoporous WO_3_-25 NFs is higher than the non-mesoporous WO_3_ NFs during the operating temperature range from 150 to 400°C (Figure 4B), and the sensitivity of mesoporous WO_3_-25 NFs is 66% higher than that of non-mesoporous WO_3_ for 50 ppm at the optimal operation temperature (Figure 4C). Moreover, the mesoporous WO_3_-25 NFs shows a response time of 24 s, much shorter than that of non-mesoporous WO_3_ (67 s), as shown in the Figure S7. The faster response is mainly attributed to its porous structure, which could facilitate the diffusion and transport of target gas via enormous pore channels to interact with WO_3_ NFs, quickly reaching the maximum sensitivity. The reversible cycles of the response curves illustrate a stable and reliable operation of acetone sensing of all the WO_3_ NFs, and further confirm the consistency of mesoporous WO_3_-25 NFs based sensors (Figure 4C). The continuous dynamic electrical response of mesoporous WO_3_-25 NFs toward different concentration of acetone (5–125 ppm) at 300°C is shown in Figure 5A. With the increase in acetone concentration, the response values of mesoporous WO_3_-25 NFs based gas sensors rapidly increased from 3.1 at 5 ppm to 89 at 125 ppm. After aeration, the acetone molecules desorbed immediately from the surface of tungsten oxides. The response can be recovered to its initial value for all the testing concentrations, reflecting a good reversibility of the gas sensor. Surprisingly, the minimum detectable concentration can reach as low as 1 ppm (Figure S8). As shown in Figure 5B, a linear relationship between the sensing response and acetone concentrations is observed, indicating the feasibility of quantitative acetone detection by the mesoporous WO_3_ NFs based sensors. As important parameters for a gas sensor, the response and recovery behaviors are also crucial for evaluating the sensing performance. The mesoporous WO_3_-25 NFs based sensor exhibits a fast response of 24 s upon exposure to 50 ppm acetone and quick recovery of 27 s when acetone gas was removed (Figure 5C). In addition, compared with earlier reported acetone sensors, our mesoporous WO_3_ NFs exhibits much better comprehensive sensing performance such as high sensitivity, low limits of detection and fast response-recovery (Table 1), thus becoming a promising candidate for acetone sensing in environmental monitoring and rapid medical diagnosis. Based on the aforementioned results, the predominant enhancement could mainly be explained as follows: (1) The unique hierarchical structure where uniform and controllable mesopores are well-connected with enormous transportation channels derived from PVP species, which facilitate the rapid diffusion of gas molecules. (2) The high specific surface area with abundant active sites enables the adsorption of a large amount of acetone molecules. (3) The continuous crystalline framework is also favorable for the fast transportation of charge carriers from the surface into bulk. It can therefore be concluded that the unique feature of our materials can maximize the performance of gas sensing. The selectivity of gas sensors is also an important parameter in practical applications. In this work, four kinds of typical vaporous molecules with identical concentration of 50 ppm, such as methanol, ethanol, toluene, and formaldehyde were selected as interfering gases. As illustrated in Figure 5D, the response value of a mesoporous WO_3_-25 NFs based sensor to acetone was at least four times higher than that of interfering gases, which implies an excellent selectivity.

**Table 1:** Comparison of acetone sensing properties of WO_3_ or WO_3_ based sensors with various nanostructures.

**Structures**	**Working temperature**	**Acetone concentration (ppm)**	**Response (*R*_**a**_/*R*_**g**_)**	**Response/recovery time**	**References**
Porous WO_3_	300°C	50	12.1	4/11.7 s	Dong et al., 2016
WO_3_ plate	307°C	100	~15	10/26 s	Liu et al., 2012
WO_3_ nanoflowers	300°C	100	~7	Not mentioned	Wang et al., 2013
Co_3_O_4_-WO_3_ nanocomposite	280°C	100	5.3	Not mentioned	Zhao et al., 2015
Ag-WO3 nanosheets	340°C	50	~8	28/38 s	Yin et al., 2017
MoO_3_-WO_3_ nanostructures	320°C	100	18.2	8/7 s	Sun et al., 2017
WO_3_@CuO nanostructures	Not mentioned	50	3.4	72.2/29.4 s	Yang F. et al., 2018
cactus-like WO_3_-SnO_2_	360°C	100	12.1	Not mentioned	Zhu et al., 2018
Gd doped WO_3_-RGO nanostructures	350°C	50	27.0	Not mentioned	Kaur et al., 2018
La_2_O_3_-WO_3_ nanofibers	350°C	100	12.7	6/210 s	Feng et al., 2015
Mesoporous WO_3_ NFs	350°C	50	22.1	24/27 s	This wok

In summary, mesoporous WO_3_ NFs with controllable pore diameters were synthesized via a facile electrospinning of an aqueous solution containing ammonium paratungstate, PVP and SiO_2_ NPs sacrificing templates, followed by controlled pyrolysis at a high temperature and selective etching with hydrofluoric acid. The PVP species can provide rigid support inside frameworks, which can prevent the collapse of the unique fiber morphology and the well-connected porous structure. The decomposition of the PVP species during calcination and the etching of SiO_2_ NPs was also found to contribute to the formation of interconnected transportation channels and uniform mesopores in the framework of the fibers. The obtained mesoporous WO_3_ NFs possess a tunable pore size (26.3 and 42.2 nm), high surface area and pore volume (up to 34.4 m^2^/g and 0.24 cm^3^g^−1^), and a well-developed hierarchical porous structure and crystalline pore walls. Sensors based on these materials were found to have an excellent acetone sensing performance with a fast response (24 s) and recovery (27 s), a low detection limit of 1 ppm, excellent selectivity and good stability, due to their high mesoporosity, abundant active sites and the continuous crystalline framework. Based on the above-mentioned results, the obtained mesoporous WO_3_ NFs holds great promise for applications in various fields such as portable miniaturized devices used for environmental monitoring, and breath analysis used for disease pre-diagnosis and home security. Moreover, it is expected that the facile and effective electrospinning approach may open up new opportunities for the design of various mesoporous metal oxide NFs with high surface areas, crystalline frameworks and greatly improved mass diffusion and transportation for application in sensors, catalysis, energy storage and conversion.

## Author Contributions

WL and JY conceived and designed the experiments. HX, TZ, YZ and JG performed the experiments. ML, GZ, LW, WJ, YF, MZ analyzed the data. WL and HX wrote the manuscript. XQ designed the scheme. All authors reviewed the manuscript and approved the final version.

### Conflict of Interest Statement

The authors declare that the research was conducted in the absence of any commercial or financial relationships that could be construed as a potential conflict of interest.

## References

[B1] Barr T. J. Sampaio R. N. Dimarco B. N. James E. M. Meyer G. J. (2017). Phantom electrons in mesoporous nanocrystalline SnO_2_ thin films with cation-dependent reduction onsets. Chem. Mater. 29, 3919–3927. 10.1021/acs.chemmater.6b05470

[B2] Barsan N. Weimar U. (2001). Conduction model of metal oxide gas sensors. J. Electroceram. 7, 143–167. 10.1023/A:1014405811371

[B3] Channei D. Nakaruk A. Khanitchaidecha W. Jannoey P. Phanichphant S. (2018). Adsorption and photocatalytic processes of mesoporous SiO_2_-coated monoclinic BiVO_4_. Front. Chem. 6:415. 10.3389/fchem.2018.00415 30283773 PMC6156254

[B4] Chen P. Y. Dang X. Klug M. T. Courchesne N. M. D. Qi J. Hyder M. N. (2015). M13 Virus-enabled synthesis of titanium dioxide nanowires for tunable mesoporous semiconducting networks. Chem. Mater. 27, 1531–1540. 10.1021/cm503803u

[B5] Dam D. T. Lee J. M. (2013). Capacitive behavior of mesoporous manganese dioxide on indium–tin oxide nanowires. Nano Energy 2, 933–942. 10.1016/j.nanoen.2013.03.014

[B6] Dasog M. Yang Z. Veinot J. G. C. (2012). Size-controlled solid state synthesis of luminescent silicon nanocrystals using Stöber silica particles. Cryst. Eng. Comm. 14:7576 10.1039/c2ce25950h

[B7] Dong C. Liu X. Guan H. Chen G. Xiao X. Djerdj I. (2016). Combustion synthesized hierarchically porous WO_3_ for selective acetone sensing. Mater. Chem. Phys. 184, 155–161. 10.1016/j.matchemphys.2016.09.036

[B8] Du J. Lai X. Yang N. Zhai J. Kisailus D. Su F. (2011). Hierarchically ordered macro–mesoporous TiO_2_-graphene composite films: improved mass transfer, reduced charge recombination, and their enhanced photocatalytic activities. ACS Nano 5, 590–596. 10.1021/nn102767d 21189003

[B9] Feng C. Wang C. Cheng P. Li X. Wang B. Guan Y. (2015). Facile synthesis and gas sensing properties of La_2_O_3_-WO_3_ nanofibers. Sensors Actuators B Chem. 221, 434–442. 10.1016/j.snb.2015.06.114

[B10] Guo P. Zhao G. Chen P. Lei B. Jiang L. Zhang H. (2014). Porphyrin nanoassemblies via surfactant-assisted assembly and single nanofiber nanoelectronic sensors for high-performance H_2_O_2_ vapor sensing. ACS Nano 8, 3402–3411. 10.1021/nn406071f 24654963

[B11] He B. Wang J. Fan Y. Jiang Y. Zhai Y. Wang Y. (2018). Mesoporous CoO/Co–N–C nanofibers as efficient cathode catalysts for Li–O_2_ batteries. J. Mater. Chem. A 6, 19075–19084. 10.1039/C8TA07185C

[B12] Jeong Y. J. Koo W. T. Jang J. S. Kim D. H. Cho H. J. Kim I. D. (2018). Chitosan-templated Pt nanocatalyst loaded mesoporous SnO_2_ nanofibers: a superior chemiresistor toward acetone molecules. Nanoscale 10, 13713–13721. 10.1039/C8NR03242D 29989640

[B13] Jha G. Tran T. Qiao S. Ziegler J. M. Ogata A. F. Dai S. (2018). Electrophoretic deposition of mesoporous Niobium(V)oxide nanoscopic films. Chem. Mater. 30, 6549–6558. 10.1021/acs.chemmater.8b03254

[B14] Jha R. K. Wan M. Jacob C. Guha P. K. (2018). Ammonia vapour sensing properties of *in situ* polymerized conducting PANI-nanofiber/WS_2_ nanosheet composites. N. J. Chem. 42, 735–745. 10.1039/C7NJ03343E

[B15] Jia Q. Ji H. Zhang Y. Chen Y. Sun X. Jin Z. (2014). Rapid and selective detection of acetone using hierarchical ZnO gas sensor for hazardous odor markers application. J. Hazar. Mater. 276, 262–270. 10.1016/j.jhazmat.2014.05.044 24892776

[B16] Kaneti Y. V. Salunkhe R. R. Wulan Septiani N. L. Young C. Jiang X. He Y. B. (2018). General template-free strategy for fabricating mesoporous two-dimensional mixed oxide nanosheets via self-deconstruction/reconstruction of monodispersed metal glycerate nanospheres. J. Mater. Chem. A 6, 5971–5983. 10.1039/C8TA00008E

[B17] Kaur J. Anand K. Kaur A. Singh R. C. (2018). Sensitive and selective acetone sensor based on Gd doped WO_3_/reduced graphene oxide nanocomposite. Sensors Actuators B Chem. 258, 1022–1035. 10.1016/j.snb.2017.11.159

[B18] Kawano T. Chiamori H. C. Suter M. Zhou Q. Sosnowchik B. D. Lin L. (2007). An electrothermal carbon nanotube gas sensor. Nano Lett. 7, 3686–3690. 10.1021/nl071964s 18001108

[B19] Kim H. J. Lee J. H. (2014). Highly sensitive and selective gas sensors using p-type oxide semiconductors: overview. Sensors Actuators B Chem. 192, 607–627. 10.1016/j.snb.2013.11.005

[B20] Kim S. J. Choi S. J. Jang J. S. Kim N. H. Hakim M. Tuller H. L.. (2016a). Mesoporous WO_3_ nanofibers with protein-templated nanoscale catalysts for detection of trace biomarkers in exhaled breath. ACS Nano 10, 5891–5899. 10.1021/acsnano.6b01196 27166639

[B21] Li F. Chen L. Knowles G. P. Macfarlane D. R. Zhang J. (2017). Hierarchical mesoporous SnO_2_ nanosheets on carbon cloth: a robust and flexible electrocatalyst for CO_2_ reduction with high efficiency and selectivity. Angewandte Chem Int. Edn. 56, 505–509. 10.1002/anie.201608279 27925360

[B22] Li X. Ding K. Gao B. Li Q. Li Y. Fu J. (2017). Freestanding carbon encapsulated mesoporous vanadium nitride nanowires enable highly stable sulfur cathodes for lithium-sulfur batteries. Nano Energy 40, 655–662. 10.1016/j.nanoen.2017.09.018

[B23] Li Y. Luo W. Qin N. Dong J. Wei J. Li W.. (2014). Highly ordered mesoporous tungsten oxides with a large pore size and crystalline framework for H_2_S sensing. Angewandte Chem. Int. Edn. 53, 9035–9040. 10.1002/anie.201403817 24990323

[B24] Liu S. Zhang F. Li H. Chen T. Wang Y. (2012). Acetone detection properties of single crystalline tungsten oxide plates synthesized by hydrothermal method using cetyltrimethyl ammonium bromide supermolecular template. Sensors Actuators B Chem. 162, 259–268. 10.1016/j.snb.2011.12.076

[B25] Luo W. Li Y. Dong J. Wei J. Xu J. Deng Y.. (2013). A resol-assisted co-assembly approach to crystalline mesoporous niobia spheres for electrochemical biosensing. Angewandte Chem. Int. Edn. 52, 10505–10510. 10.1002/anie.201303353 23943495

[B26] Luo W. Wang Y. Wang L. Jiang W. Chou S. L. Dou S. X.. (2016a). Silicon/mesoporous carbon/crystalline TiO_2_ nanoparticles for highly stable lithium storage. ACS Nano 10, 10524–10532. 10.1021/acsnano.6b06517 27786460

[B27] Luo W. Zhao T. Li Y. Wei J. Xu P. Li X.. (2016b). A Micelle fusion-aggregation assembly approach to mesoporous carbon materials with rich active sites for ultrasensitive ammonia sensing. J. Am. Chem. Soc. 138, 12586–12595. 10.1021/jacs.6b07355 27575996

[B28] Ma J. Ren Y. Zhou X. Liu L. Zhu Y. Cheng X. (2018). Pt Nanoparticles sensitized ordered mesoporous WO_3_ semiconductor: gas sensing performance and mechanism study. Adv. Funct. Mater. 28:1705268 10.1002/adfm.201705268

[B29] Nada A. A. Nasr M. Viter R. Miele P. Roualdes S. Bechelany M. (2017). Mesoporous ZnFe_2_O_4_@TiO_2_ nanofibers prepared by electrospinning coupled to PECVD as highly performing photocatalytic materials. J. Phys. Chem. C 121, 24669–24677. 10.1021/acs.jpcc.7b08567

[B30] Qiu Z. Hua Z. Li Y. Wang M. Huang D. Tian C.. (2018). Acetone sensing properties and mechanism of Rh-Loaded WO_3_ nanosheets. Front. Chem. 6, 385–385. 10.3389/fchem.2018.00385 30255011 PMC6141622

[B31] Rakhi R. B. Chen W. Cha D. Alshareef H. N. (2012). Substrate dependent self-organization of mesoporous cobalt oxide nanowires with remarkable pseudocapacitance. Nano Lett. 12, 2559–2567. 10.1021/nl300779a 22494065

[B32] Ren X. Hou H. Liu Z. Gao F. Zheng J. Wang L.. (2016). Shape-enhanced photocatalytic activities of thoroughly mesoporous ZnO nanofibers. Small 12, 4007–4017. 10.1002/smll.201600991 27337544

[B33] Saha J. De G. (2013). Highly ordered cubic mesoporous electrospun SiO_2_ nanofibers. Chem. Commun. 49, 6322–6324. 10.1039/c3cc42338g 23743484

[B34] Salehi S. Nikan E. Khodadadi A. A. Mortazavi Y. (2014). Highly sensitive carbon nanotubes–SnO_2_ nanocomposite sensor for acetone detection in diabetes mellitus breath. Sensors Actuators B Chem. 205, 261–267. 10.1016/j.snb.2014.08.082

[B35] Shi L. Wang W. Wang A. Yuan K. Jin Z. Yang Y. (2016). Scalable synthesis of core-shell structured SiO_x_/nitrogen-doped carbon composite as a high-performance anode material for lithium-ion batteries. J. Power Sources 318, 184–191. 10.1016/j.jpowsour.2016.03.111

[B36] Singkammo S. Wisitsoraat A. Sriprachuabwong C. Tuantranont A. Phanichphant S. Liewhiran C. (2015). Electrolytically exfoliated graphene-loaded flame-made Ni-doped SnO_2_ composite film for acetone sensing. ACS Appl. Mater. Interfaces 7, 3077–3092. 10.1021/acsami.5b00161 25602118

[B37] Sun Y. Chen L. Wang Y. Zhao Z. Li P. Zhang W. (2017). Synthesis of MoO_3_/WO_3_ composite nanostructures for highly sensitive ethanol and acetone detection. J. Mater. Sci. 52, 1561–1572. 10.1007/s10853-016-0450-2

[B38] Wang C. Hashimoto T. (2018). Self-organization in electrospun polymer solutions: from dissipative structures to ordered fiber structures through fluctuations. Macromolecules 51, 4502–4515. 10.1021/acs.macromol.8b00647

[B39] Wang C. Yin L. Zhang L. Xiang D. Gao R. (2010). Metal oxide gas sensors: sensitivity and influencing factors. Sensors 10, 2088–2106. 10.3390/s100302088 22294916 PMC3264469

[B40] Wang C. Y. Zhang X. Rong Q. Hou N. N. Yu H. Q. (2018). Ammonia sensing by closely packed WO_3_ microspheres with oxygen vacancies. Chemosphere 204, 202–209. 10.1016/j.chemosphere.2018.04.050 29656156

[B41] Wang G. Ling Y. Wang H. Yang X. Wang C. Zhang J. Z. (2012). Hydrogen-treated WO_3_ nanoflakes show enhanced photostability. Energy Environ. Sci. 5, 6180–6187. 10.1039/c2ee03158b

[B42] Wang L. Bi X. Yang S. (2016). Partially single-crystalline mesoporous Nb_2_O_5_ nanosheets in between graphene for ultrafast sodium storage. Adv. Mater. 28, 7672–7679. 10.1002/adma.201601723 27346391

[B43] Wang M. Fan L. Wu X. Tian D. Cheng J. Qiu Y. (2017). Hierarchical mesoporous SnO_2_ nanosheets on carbon cloth toward enhancing the polysulfides redox for lithium–sulfur batteries. J. Mater. Chem. A 5, 19613–19618. 10.1039/C7TA04937D

[B44] Wang S. Ang H. M. Tade M. O. (2007). Volatile organic compounds in indoor environment and photocatalytic oxidation: state of the art. Environ. Int. 33, 694–705. 10.1016/j.envint.2007.02.011 17376530

[B45] Wang W. Q. Wang X. L. Xia X. H. Yao Z. J. Zhong Y. Tu J. P. (2018). Enhanced electrochromic and energy storage performance in mesoporous WO_3_ film and its application in a bi-functional smart window. Nanoscale 10, 8162–8169. 10.1039/C8NR00790J 29676415

[B46] Wang X. D. Summers C. J. Wang Z. L. (2004). Mesoporous single-crystal ZnO nanowires epitaxially sheathed with Zn_2_SiO_4_. Adv. Mater. 16, 1215–1218. 10.1002/adma.200306505

[B47] Wang Z. Sun P. Yang T. Gao Y. Li X. Lu G. (2013). Flower-like WO_3_ architectures synthesized via a microwave-assisted method and their gas sensing properties. Sensors Actuators B Chem. 186, 734–740. 10.1016/j.snb.2013.06.015

[B48] Xu K. Ben L. Li H. Huang X. (2015). Silicon-based nanosheets synthesized by a topochemical reaction for use as anodes for lithium ion batteries. Nano Res. 8, 2654–2662. 10.1007/s12274-015-0772-4

[B49] Yamazoe N. Sakai G. Shimanoe K. (2003). Oxide Semiconductor Gas Sensors. Catalysis Surveys Asia 7, 63–75. 10.1023/A:1023436725457

[B50] Yan S. Xue J. Wu Q. (2018). Synchronous synthesis and sensing performance of α-Fe_2_O_3_/SnO_2_ nanofiber heterostructures for conductometric C_2_H_5_OH detection. Sensors Actuators B Chem. 275, 322–331. 10.1016/j.snb.2018.07.079

[B51] Yang F. Wang F. Guo Z. (2018). Characteristics of binary WO_3_@CuO and ternary WO_3_@PDA@CuO based on impressive sensing acetone odor. J. Colloid Interface Sci. 524, 32–41. 10.1016/j.jcis.2018.04.013 29627670

[B52] Yang Z. Huang Y. Yao F. Luo H. Wan Y. (2018). Wrapping mesoporous Fe_2_O_3_ nanoparticles by reduced graphene oxide: enhancement of cycling stability and capacity of lithium ion batteries by mesoscopic engineering. Ceramics Int. 44, 20656–20663. 10.1016/j.ceramint.2018.08.058

[B53] Yin M. Yu L. Liu S. (2017). Synthesis of Ag quantum dots sensitized WO_3_ nanosheets and their enhanced acetone sensing properties. Mater. Lett. 186, 66–69. 10.1016/j.matlet.2016.09.083

[B54] Yoon J. Yang H. S. Lee B. S. Yu W. R. (2018). Recent progress in coaxial electrospinning: new parameters, various structures, and wide applications. Adv. Mater. 30:e1704765. 10.1002/adma.201704765 30152180

[B55] Zhang D. Zhang N. Ma F.-F. Qi X. D. Yang J. H. Huang T.. (2018). One-step fabrication of functionalized poly(l-lactide) porous fibers by electrospinning and the adsorption/separation abilities. J. Hazard. Mater. 360, 150–162. 10.1016/j.jhazmat.2018.07.090 30099358

[B56] Zhang H. Li H. Wang Z. Zheng Z. Wang P. Liu Y. (2018). Fabrication of BiVO_4_ photoanode consisted of mesoporous nanoparticles with improved bulk charge separation efficiency. Appl. Catalysis B Environ. 238, 586–591. 10.1016/j.apcatb.2018.07.050

[B57] Zhang Q. Zhang H. Xu M. Shen Z. Wei Q. (2018). A WO_3_ nanorod-Cr_2_O_3_ nanoparticle composite for selective gas sensing of 2-butanone. Chin. Chem. Lett. 29, 538–542. 10.1016/j.cclet.2017.09.018

[B58] Zhang X. Dong Z. Liu S. Shi Y. Dong Y. Feng W. (2017). Maize straw-templated hierarchical porous ZnO:Ni with enhanced acetone gas sensing properties. Sensors Actuators B Chem. 243, 1224–1230. 10.1016/j.snb.2016.12.076

[B59] Zhang Y. Xu J. Xiang Q. Li H. Pan Q. Xu P. (2009). Brush-like hierarchical ZnO nanostructures: synthesis, photoluminescence and gas sensor properties. J. Phys. Chem. C 113, 3430–3435. 10.1021/jp8092258

[B60] Zhang Y. Yue Q. Yu L. Yang X. Hou X. F. Zhao D.. (2018). Amphiphilic block copolymers directed interface coassembly to construct multifunctional microspheres with magnetic core and monolayer mesoporous aluminosilicate shell. Adv. Mater. 30:1800345. 10.1002/adma.201800345 29749031

[B61] Zhao T. Luo W. Deng Y. Luo Y. Xu P. Liu Y. (2016). Monodisperse mesoporous TiO_2_ microspheres for dye sensitized solar cells. Nano Energy 26, 16–25. 10.1016/j.nanoen.2016.04.050

[B62] Zhao X. Ji H. Jia Q. Wang M. (2015). A nanoscale Co_3_O_4_-WO_3_ p–n junction sensor with enhanced acetone responsivity. J. Mater. Sci. Mater. Electron. 26, 8217–8223. 10.1007/s10854-015-3484-3

[B63] Zhao Y. Dong F. Han W. Zhao H. Tang Z. (2019). The synergistic catalytic effect between graphene oxide and three-dimensional ordered mesoporous Co_3_O_4_ nanoparticles for low-temperature CO oxidation. Microporous Mesoporous Mater. 273, 1–9. 10.1016/j.micromeso.2018.06.042

[B64] Zhou X. Cheng X. Zhu Y. Elzatahry A. A. Alghamdi A. Deng Y. (2018). Ordered porous metal oxide semiconductors for gas sensing. Chin. Chem. Lett. 29, 405–416. 10.1016/j.cclet.2017.06.021

[B65] Zhu L. Zeng W. Li Y. (2018). A novel cactus-like WO_3_-SnO_2_ nanocomposite and its acetone gas sensing properties. Mater. Lett. 231, 5–7. 10.1016/j.matlet.2018.08.007

[B66] Zhu Y. Zhao Y. Ma J. Cheng X. Xie J. Xu P.. (2017a). Mesoporous tungsten oxides with crystalline framework for highly sensitive and selective detection of foodborne pathogens. J. Am. Chem. Soc. 139, 10365–10373. 10.1021/jacs.7b04221 28683546

