# Supplementary material for: Mesoporous WO3 Nanofibers With Crystalline Framework for High-Performance Acetone Sensing
Source: Front Chem. 2019 Apr 18;7:266. doi: 10.3389/fchem.2019.00266 (PMC6482242; doi:10.3389/fchem.2019.00266)
Supplement: Supplementary file 1 [file Table_1.DOCX]

**Mesoporous WO_3_ Nanofibers with Crystalline Framework for High-Performance Acetone Sensing**

Haiyun Xu^1†^, Jie Gao^1†^, Minhan Li^1^, Yuye Zhao^1^, Ming Zhang^2^, Tao Zhao^1^, Lianjun Wang^1^, Wan Jiang^1,3,4^, Guanjia Zhu^1^, Xiaoyong Qian^1^, Yuchi Fan^3^, Jianping Yang^1,3^*, Wei Luo^1,3^*

^1^State Key Laboratory for Modification of Chemical Fibers and Polymer Materials, College of Materials Science and Engineering, Donghua University, Shanghai 201620, China

^2^Materials Genome Institute, Shanghai University, Shanghai 200444, China

^3^Institute of Functional Materials, Donghua University, Shanghai 201620, China

^4^School of Materials Science and Engineering, Jingdezhen Ceramic Institute, Jingdezhen 333001, Jiangxi, China

^†^These authors have contributed equally to this work

* Correspondence:

Jianping Yang, jianpingyang@dhu.edu.cn

Wei Luo, wluo@dhu.edu.cn


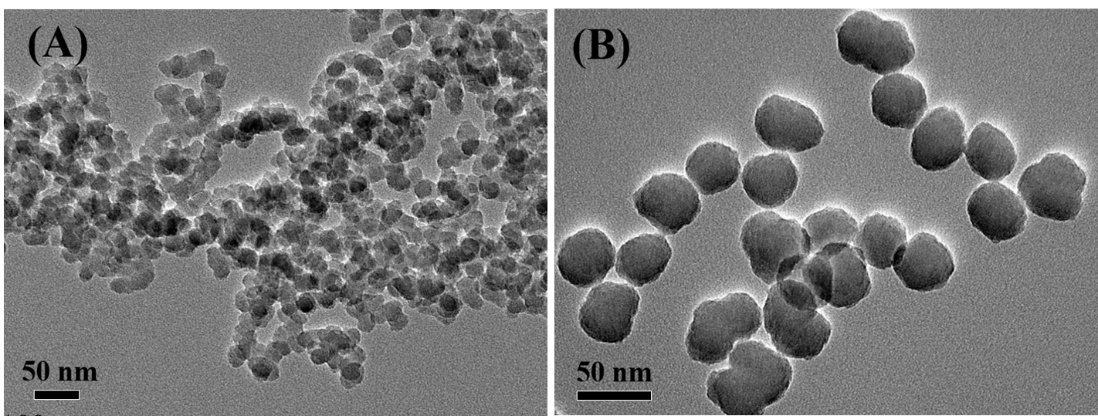


**FIGURE S1** TEM images of SiO_2_ nanoparticles with diameters of: **(A)** 25 nm and **(B)** 40nm, respectively


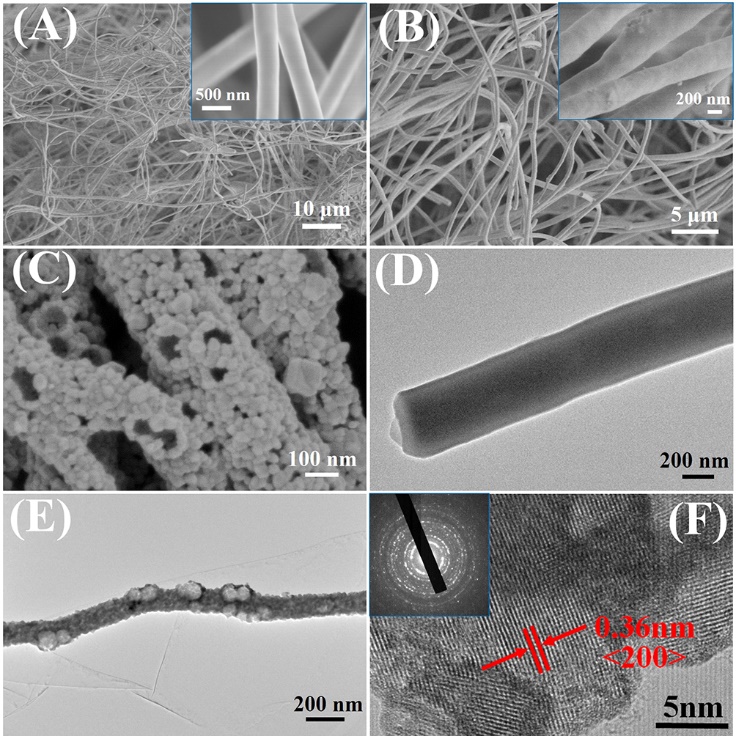


**FIGURE S2** FESEM images of **(A)** as-spun tungsten species/PVP/SiO_2_ (40 nm) NFs, **(B)** WO_3_/carbon/SiO_2_ (40 nm) NFs, **(C)** mesoporous WO_3_-40 NFs. TEM images of **(D)** WO_3_/carbon/SiO_2_ (40 nm) NFs, **(E)** mesoporous WO_3_-40NFs. HRTEM image of **(F)** mesoporous WO_3_-40 NFs.


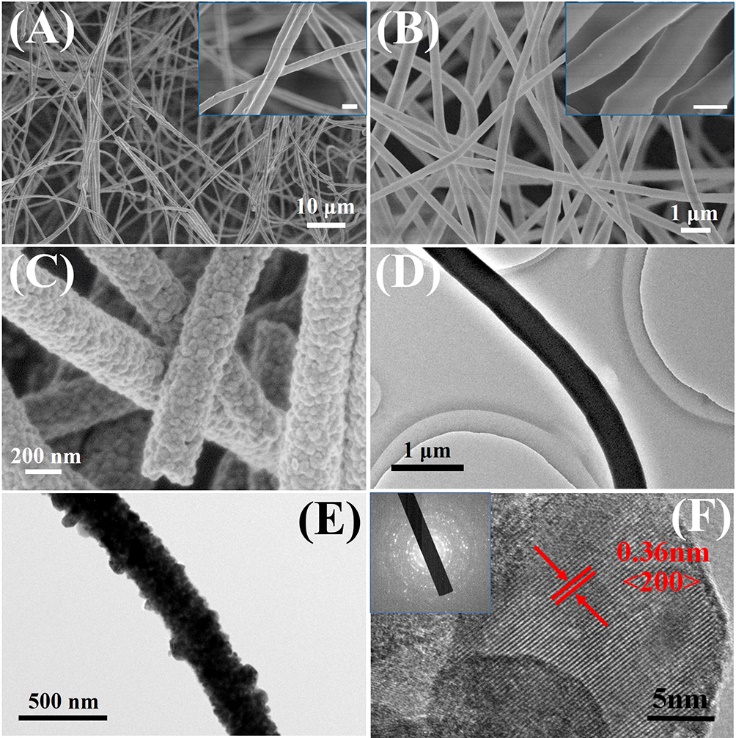


**FIGURE S3** FESEM images of **(A)** as-spun tungsten species/PVP NFs, **(B)** WO_3_/carbon NFs, **(C)** non-mesoporous WO_3_ NFs. TEM images of **(D)** WO_3_/carbon NFs, **(E)** non-mesoporous WO_3_ NFs. HRTEM image of **(F)** non-mesoporous WO_3_ NFs.


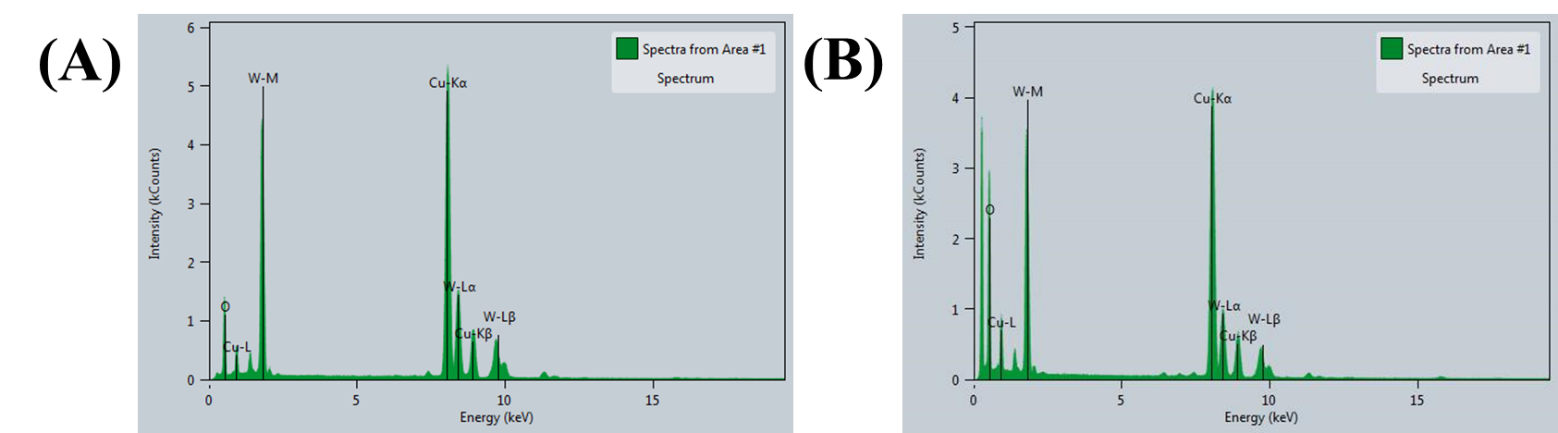


**FIGURE S4** EDS spectrum of **(A)** mesoporous WO_3_-25 NFs and **(B)** mesoporous WO_3_-40 NFs.


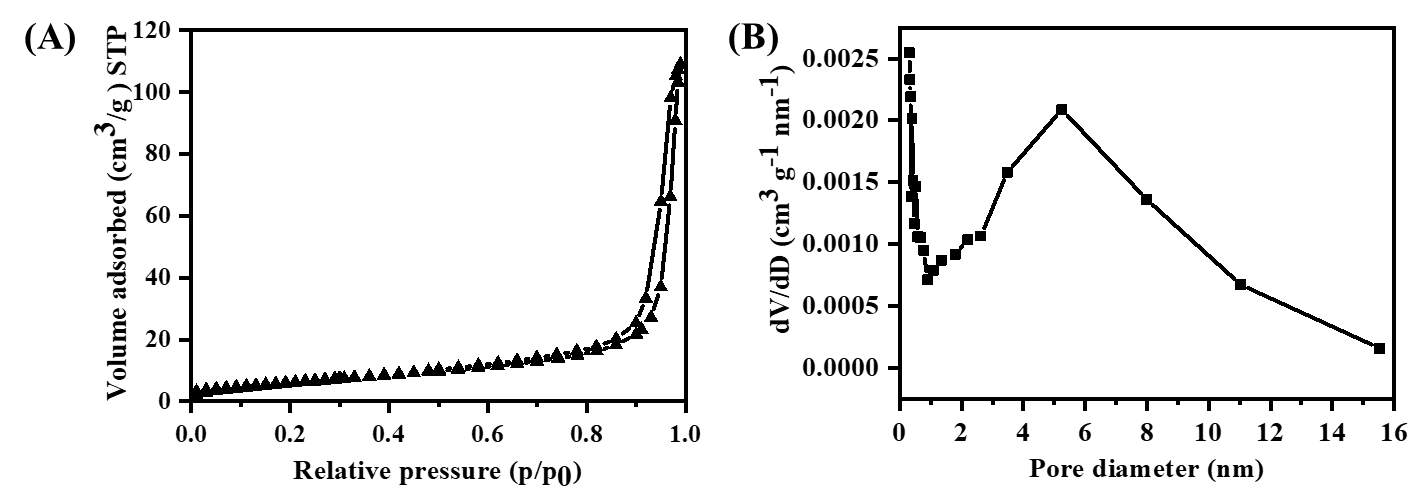


**FIGURE S5 (A)** Nitrogen adsorption-desorption isotherm and **(B)** pore size distribution of non-mesoporous WO_3_ NFs.


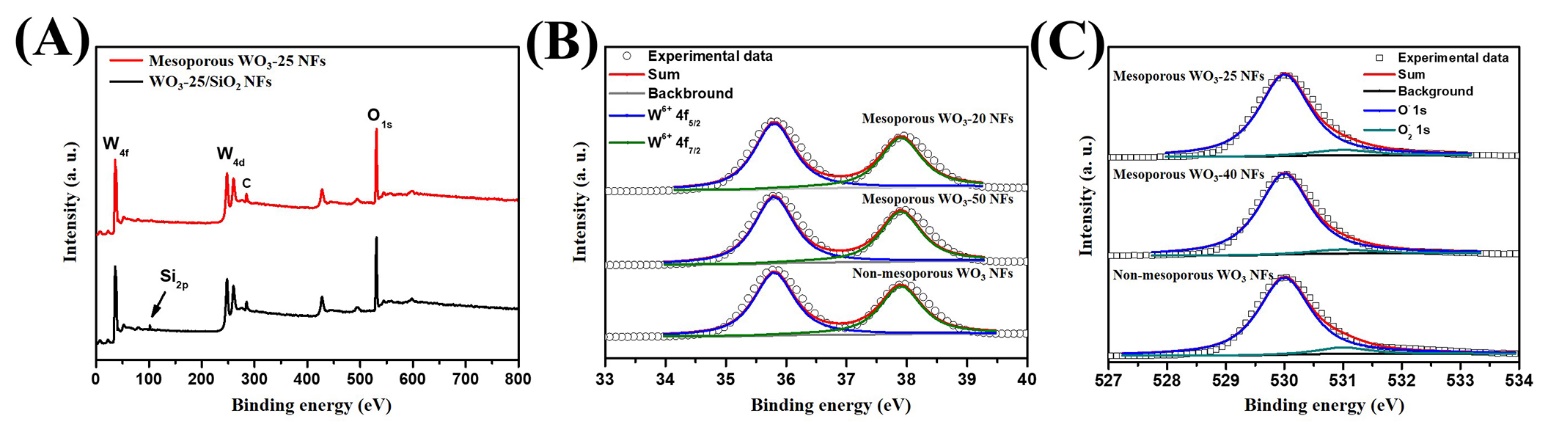


**FIGURE S6 (A)** XPS survey scans of WO_3_-25/SiO_2_ NFs and mesoporous WO_3_-25 NFs. XPS spectra of mesoporous WO_3_-25 NFs, mesoporous WO_3_-40 NFs and non-mesoporous WO_3_ NFs in the vicinity of **(B)** W and **(C)** O.


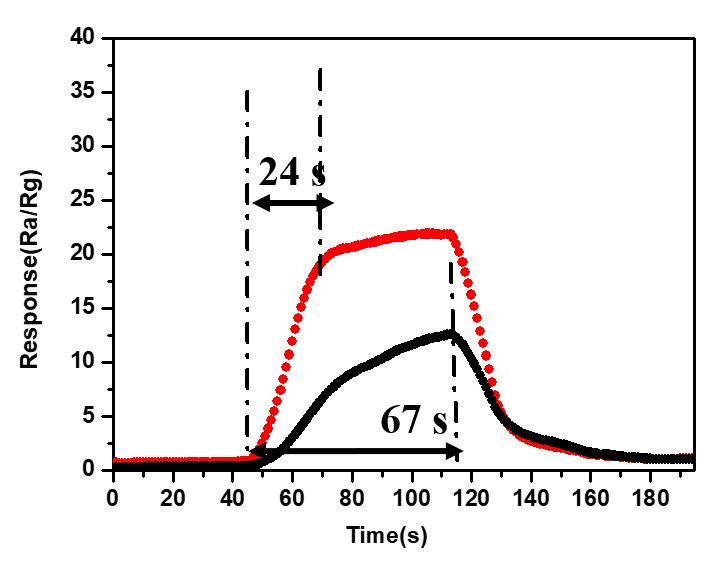


**FIGURE S7** Dynamic response curve of mesoporous WO_3_-25 NFs and non-mesoporous WO_3_ NFs sensor to 50 ppm acetone.


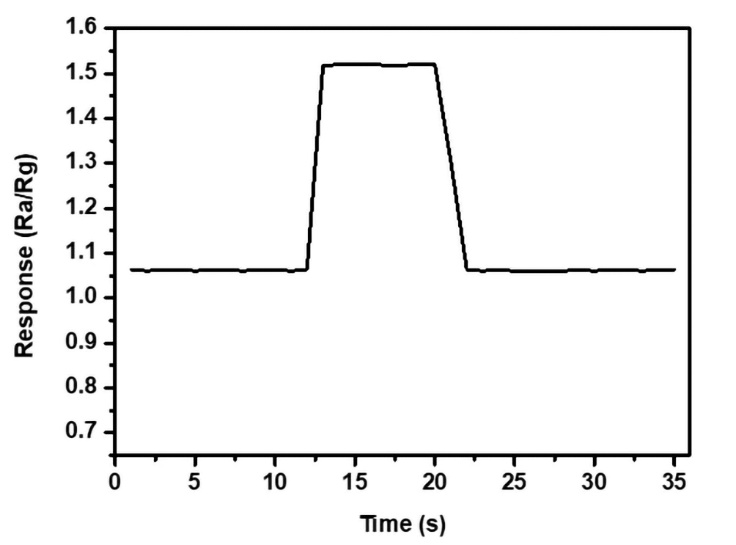


**FIGURE S8** Mesoporous WO_3_-25 NFs toward 1ppm of acetone at 300 ^o^C
